# Complete Genome Sequences of Four Halophilic Thermus thermophilus Strains Isolated from Arima Hot Spring in Japan

**DOI:** 10.1128/MRA.00874-21

**Published:** 2021-10-07

**Authors:** Kentaro Miyazaki, Natsuki Tomariguchi, Yuko Ueno

**Affiliations:** a Bioproduction Research Institute, National Institute of Advanced Industrial Science and Technology (AIST), Tsukuba, Ibaraki, Japan; b Department of Computational Biology and Medical Sciences, Graduate School of Frontier Sciences, The University of Tokyo, Kashiwa, Chiba, Japan; c Faculty of Life Sciences, Toyo University, Itakura, Gunma, Japan; d Frontier Development Division, Leave a Nest Co., Ltd., Shinjuku, Tokyo, Japan; University of Delaware

## Abstract

We isolated four Thermus thermophilus strains from Arima Hot Spring in Japan. Complete genome sequencing revealed that they showed average nucleotide identities of ≥99.21% to each other and to strains previously isolated from the same spot, but of ≤97.86% to strains from geographically different spots in Japan, reflecting habitat-specific genomic conservation.

## ANNOUNCEMENT

Thermus thermophilus, which grows optimally at around 70 to 75°C, was first isolated from Mine Hot Spring in Japan in 1968 (1, 2). So far, complete genome sequences have been reported for 13 strains (https://www.ncbi.nlm.nih.gov/genome/browse#!/prokaryotes/461/), of which six were isolated from Mine Hot Spring. Comparative genomic analyses (3) of these six strains (4–7) revealed high (≥98.53%) average nucleotide identities (ANIs) to each other, supporting habitat-specific genomic conservation. In this study, to gain insight into the habitat-dependent evolutionary patterns of T. thermophilus, we investigated the genomes of four T. thermophilus strains that were newly isolated from Arima Hot Spring in Japan (34.7974 N, 135.2494 E). Arima Hot Spring lies in a mountain setting; however, the hot spring water, known as Arima-type brine (8), is unique in its high salinity and high solute concentrations. For bacterial isolation, a hot water sample (pH 6.6, 88°C, ca. 42,000 ppm Cl) was spread over marine agar (Sigma) plates. After incubation at 65°C for 24 h, four well-separated colonies were isolated; repetitive colony isolation by streaking ensured axenicity. These strains, designated AA1-1, AA2-2, AA3-7, and AK1, were subjected to genome analyses.

To prepare the genomic DNA, cells were grown in 5 ml of marine broth (Sigma) at 70°C for 24 h with vigorous shaking (200 rpm). The genomic DNA was purified using a blood and cell culture DNA Midi kit (Qiagen). For long-read sequencing, unsheared genomic DNA (1 μg) was treated with a short-read eliminator kit (Circulomics) to remove fragments of <10 kbp, and a library was constructed using a ligation sequencing kit (Oxford Nanopore Technologies [ONT]). Sequencing was performed using a GridION X5 system on a FLO-MIN106 R9.41 rev D flow cell (ONT). Base calling was conducted using Guppy v.4.0.11. The raw sequencing data (Table 1) were filtered (Q < 10; length, <1,000 bases) using NanoFilt v.2.7.1 (9). For short-read sequencing, a library was constructed using an MGIEasy FS PCR-free DNA library prep set (MGI) with a ∼400 to 500-bp insert. Paired-end sequencing (2 × 150 bases) was then performed on a DNBSEQ-400 instrument (MGI). The raw sequencing data (Table 1) were filtered (Q < 30; length, <10 bases) using fastp v.0.20.1 (10). For all software, default parameters were used.

**TABLE 1:** Characteristics and accession numbers of four halophilic Thermus thermophilus genomes

Strain	DNBSEQ (short read)			GridION (long read)				Length (bp)	GC content (%)	No. of coding sequences	Avg read depth (×)	GenBank accession no.
	No. of paired-end reads	Total length (Mb)	SRA accession no.	No. of reads	*N*_50_ (bp)	Total length (Mb)	SRA accession no.					
AA1-1	7,640,627	2,292	DRR308214	151,074	26,864	1,200	DRR308215					
Chromosome								1,954,152	69.2	2,092	1,056.2	AP024926
pAA1-1b								227,954	68.3	263	684.0	AP024927
pAA1-1c								19,112	69.4	24	5,199.4	AP024928
AA2-2	6,156,709	1,847	DRR308216	602,728	9,095	2,546	DRR308217					
Chromosome								1,948,114	69.2	2,076	376.0	AP024929
pAA2-2b								298,084	68.8	327	452.1	AP024930
pAA2-2c								164,734	66.6	196	222.6	AP024931
pAA2-2d								13,273	66.7	7	1,753.2	AP024932
AA3-7	4,675,366	1,402	DRR308218	426,341	7,807	1,781	DRR308219					
Chromosome								1,980,678	69.1	2,139	273.3	AP024933
pAA3-7b								213,325	69.3	243	280.2	AP024934
pAA3-7c								143,809	68.2	72	212.9	AP024935
pAA3-7d								1,4256	67.7	8	1,419.4	AP024936
AK1	7,754,567	2,326	DRR308220	294,172	9,819	1,614	DRR308221					
Chromosome								1,946,623	69.2	2,096	767.6	AP024937
pAK1b								116,199	68.7	135	1,716.6	AP024938
pAK1c								91,082	68.2	20	497.8	AP024939

A hybrid assembly of the trimmed long- and short-read data was conducted using Unicycler v.0.4.8 (11), and the assembly was polished using Pilon v.1.23 (12). Each strain contained a single circular chromosome and two or three circular plasmids; circularity was confirmed using Unicycler. Automatic annotation was conducted using DFAST v.1.4.0 (13); the genomic features are summarized in Table 1. A JSpecies analysis (14) revealed that the genome sequences of bacterial strains from Arima Hot Spring (four strains presented in this study and two previously documented strains, AA2-20 [15] and AA2-29 [15]) showed ANIs of ≥99.21% to each other but of ≤97.86% to six strains originating from Mine Hot Spring (HB8, HB27 [4], HC11 [5], HB5002 [7], HB5008 [7], and HB5018 [6]).

### Data availability.

All four T. thermophilus strains reported in this paper are associated with BioProject accession number PRJDB7414. The genome sequences and raw sequencing data are available under the accession numbers listed in Table 1.
